# Redressing the interactions between stem cells and immune system in tissue regeneration

**DOI:** 10.1186/s13062-021-00306-6

**Published:** 2021-10-20

**Authors:** Jiankai Fang, Chao Feng, Wangwang Chen, Pengbo Hou, Zhanhong Liu, Muqiu Zuo, Yuyi Han, Chenchang Xu, Gerry Melino, Alexei Verkhratsky, Ying Wang, Changshun Shao, Yufang Shi

**Affiliations:** 1 The Third Affiliated Hospital of Soochow University, Institutes for Translational Medicine, State Key Laboratory of Radiation Medicine and Protection, Medical College of Soochow University, 199 Renai Road, Suzhou, 215123 Jiangsu People’s Republic of China; 2 Department of Experimental Medicine and Biochemical Sciences, TOR, University of Rome Tor Vergata, Rome, Italy; 3 Laboratory Animal Center, Medical College of Soochow University, Suzhou, Jiangsu People’s Republic of China; 4 Faculty of Biology, Medicine and Health, University of Manchester, Manchester, M13 9PT UK; 5 Shanghai Institute of Nutrition and Health, Shanghai Institutes for Biological Sciences, Chinese Academy of Sciences, 320 Yueyang Road, Shanghai, 200031 People’s Republic of China

**Keywords:** MuSCs, Immune cells, Immunomodulation, Muscle repair, Tissue regeneration

## Abstract

Skeletal muscle has an extraordinary regenerative capacity reflecting the rapid activation and effective differentiation of muscle stem cells (MuSCs). In the course of muscle regeneration, MuSCs are reprogrammed by immune cells. In turn, MuSCs confer immune cells anti-inflammatory properties to resolve inflammation and facilitate tissue repair. Indeed, MuSCs can exert therapeutic effects on various degenerative and inflammatory disorders based on their immunoregulatory ability, including effects primed by interferon-γ (IFN-γ) and tumor necrosis factor-α (TNF-α). At the molecular level, the tryptophan metabolites, kynurenine or kynurenic acid, produced by indoleamine 2,3-dioxygenase (IDO), augment the expression of TNF-stimulated gene 6 (TSG6) through the activation of the aryl hydrocarbon receptor (AHR). In addition, insulin growth factor 2 (IGF2) produced by MuSCs can endow maturing macrophages oxidative phosphorylation (OXPHOS)-dependent anti-inflammatory functions. Herein, we summarize the current understanding of the immunomodulatory characteristics of MuSCs and the issues related to their potential applications in pathological conditions, including COVID-19.

## Introduction

For centuries, biologists have been puzzled with the near perfect ability to regenerate severed body parts in certain animals. Planarians, for example, even after being sliced into 100 segments, demonstrate remarkable regeneration when each of the pieces develops into individual worm with normal adult structure. A salamander completely regrows amputated limbs with normal tissue structures and functions [1–4]. Mechanisms of accurate coordination of diverse and complex biological processes, including, but not limited to, angiogenesis, skeletal reconstruction, muscle layout, and nerve innervation during tissue regeneration remain largely elusive and merit further investigations.

Tissue regeneration is a highly orchestrated process that is often accompanied by inflammation. The infiltration of immune cells into the injured site is one of the earliest detectable responses to tissue damage. Various aspects of inflammation, such as the duration, severity, and the types of immune response, determine the outcomes of tissue regeneration [5–7]. Persistent deregulated inflammation at the injury site derails the regeneration process and eventually leads to the formation of tissue fibrosis and scars. Successful tissue regeneration requires an orderly coordination of activation and waning of different types of inflammatory responses mediated by distinct immune cell populations [8]. The immune aspect of tissue repair was not appreciated until Elie Metchnikoff discovered the role of macrophages in starfish wound healing in 1882 when being in Messina [9]. Numerous breakthroughs have been made over the recent two decades and laid a solid foundation for understanding the pathogenesis of traumatic diseases and for developing more efficient therapies, including the effects on redox, epigenetics, transcriptional, degradative, apoptotic and autophagic mechanisms [10–26].

Skeletal muscle is a major immunomodulatory organ [27]. In addition to myogenic cells, a large number of immune cells reside in healthy skeletal muscle tissue. There are 500 to 2000 leukocytes per mm^3^ of adult rodent limb muscles. Skeletal muscle is the largest tissue, accounting for approximately 35% of the total body mass of a human and containing about 10^9^ leukocytes per liter of muscle tissue. Total number of leukocytes in a typical adult is 4 × 10^11^, while there are 4 × 10^10^ leukocytes in the entire muscle [28]. T cells, neutrophils and eosinophils are less abundant in healthy skeletal muscle, whereas monocytes and macrophages account for the majority of resident immune cell populations. They are scattered in the interstices of muscle connective tissue and around blood vessels [29, 30]. The muscle immune system represents an excellent platform for investigating the interactions between stem cells and immune cells in tissue regeneration.

**Fig. 1:**
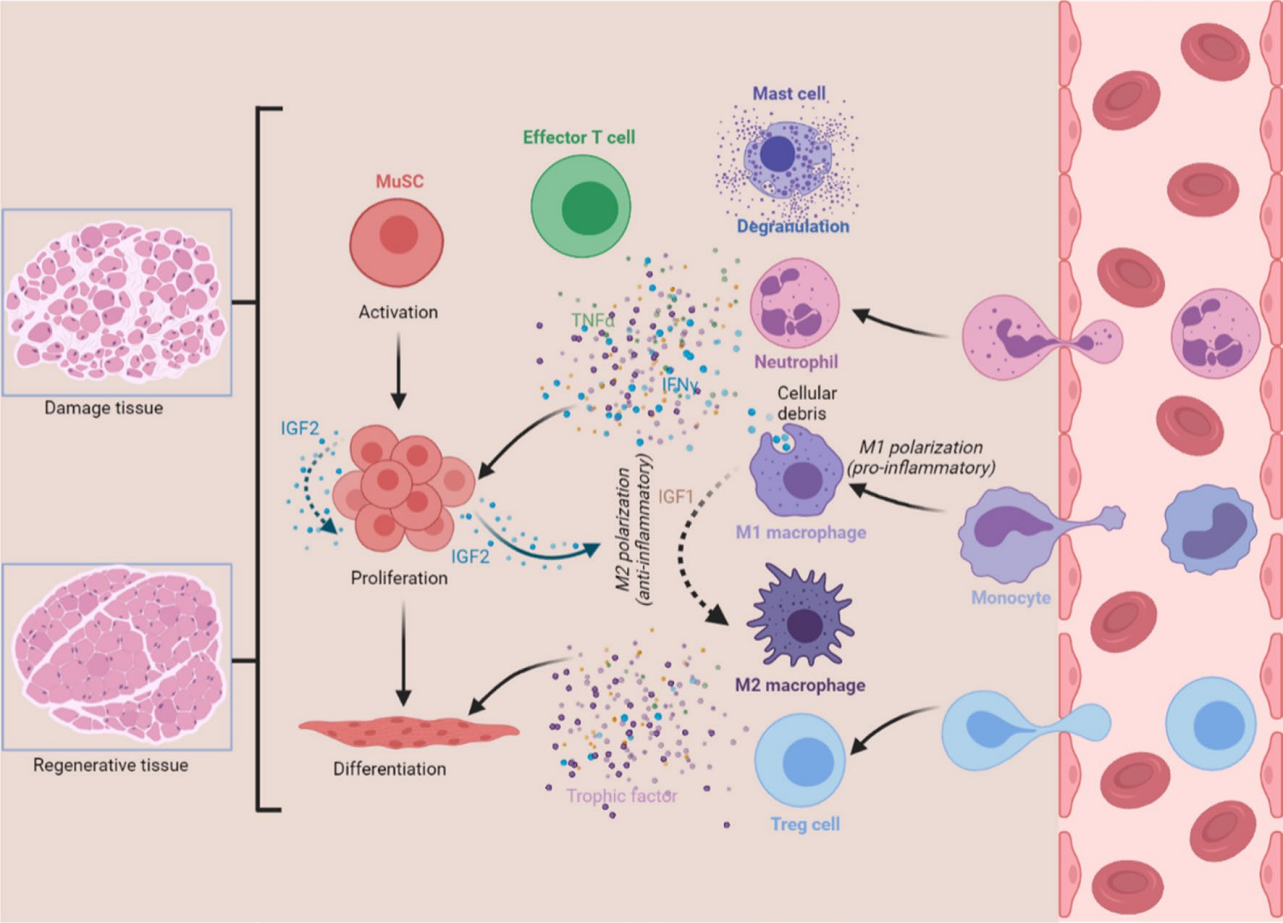
The reciprocal interaction between MuSCs and immune cells during muscle regeneration. Trauma to muscle evokes dramatic immune challenge. Within hours, damaged muscle tissue experiences mast cell degranulation, invasion by neutrophils, infiltration by M1 macrophages, and trafficking of effector T cells. These immune cells condition the inflammatory environment that is enriched with pro­inflammatory cytokines, including IFN-γ and TNF-α, and direct rapid expansion of the MuSC population. The shift of macrophage phenotype functionally couples with the transition of stages of myogenesis. IGF1 modulates autocrine polarization of pro-inflammatory macrophages towards a M2 phenotype. Treg cells also promote the transition of macrophage phenotype through paracrine action of IL-10. Furthermore, MuSCs could orchestrate inflammatory microenvironments through directing the switch towards anti-inflammatory macrophages via the action of IGF2. The resolution of inflammation through multiple mechanisms drives the later stages of MuSC differentiation. Thus, the bidirectional interaction between MuSCs and immune cells determines the course and outcome of muscle regeneration

Muscle damage caused by trauma, burns, freezing, toxins, over-exercise or certain diseases often disrupts the original tissue structure. Muscle stem cells (MuSCs; satellite cells), laying along the fully differentiated myofibers, exit quiescence state and execute the stereotypical myogenesis program in response to muscle damage. The process of recruiting MuSCs into the reparative action can be broadly divided into (i) an early stage of MuSC activation and proliferation, and (ii) a later stage of terminal differentiation and growth [28, 31]. This regenerative process is accompanied by two distinct immunological phases: (i) the pro-inflammatory phase, where leukocytes migrate towards the injured site to exert pro-inflammatory functions and govern MuSC activation and proliferation, and (ii) the anti-inflammatory or restorative phase, which is characterized by myogenic lineage differentiation, growth of new myofibers, angiogenesis, and extracellular matrix (ECM) deposition and remodeling [32, 33] (Fig. 1).

## Regulation of myogenesis by pro-inflammatory responses

Immune cells are temporally and spatially orchestrated in response to muscle injury. Complement system, the first sensor of muscle damage, is rapidly activated in the injured area [34]. Subsequently, a cellular response occurs, during which activated resident mast cells swiftly degranulate and release pro-inflammatory cytokines to recruit other immune cells to muscle lesions [35, 36]. Within hours, neutrophils, among the first wave of peripheral immune cells, extravasate from circulation and enter the injured area, clean necrotic debris, and mount a pro-inflammatory response [37]. Waves of pro-inflammatory cytokines, such as IFN-γ, IL-1β, TNF-α, IL-1 and IL-8, are secreted from neutrophils and induce the infiltration of circulating monocytes to establish the early inflammatory microenvironment and the subsequent degradation of injured tissues [38, 39]. Macrophages, the professional phagocytes of the immune system, scavenge cellular debris to prevent the persistence of potentially toxic or immunogenic materials in the muscle environment [8, 40]. The fibro/adipocyte progenitors (FAPs), activated by IL-4-derived from eosinophils, are the non-professional phagocytes that mediate rapid debris clearance [41]. Surprisingly, compared to macrophages, FAPs were four-fold more efficient at phagocytizing necrotic thymocytes obtained through iterative cycles of freeze/thaw in liquid nitrogen in vitro [41].

The muscle environment orchestrated by macrophages and enriched with pro-­inflammatory cytokines is essential for the initial stages of myogenesis. The destruction of CCL2/CCR2 axis, the main attractant of circulating monocytes into a damaged site, through genetic ablation of *Ccl2* or its receptor *Ccr2* reduced infiltrating macrophage numbers in injured muscle, and was accompanied by slowed growth of nascent muscle fibers [42–45]. Other parallel investigations employing myeloid depletion models (clodronate, *CD11b-DTR* mouse) also highlighted the absolute requirement of macrophages in governing muscle inflammation and triggering myogenic program following injury [46]. In addition, complement protein C3a has been implicated in promoting the trafficking of macrophages in damaged muscles to facilitate tissue regeneration [47].

However, infiltrating macrophages exhibit distinct phenotypic characteristics and functions in different phases of skeletal muscle regeneration [32, 48]. The dichotomy between M1 (classically activated) and M2 (alternatively activated) macrophages represents distinct polarized phenotypes, signifying the transition from a pro-inflammatory to an anti-inflammatory/restorative profile. Nevertheless, this M1-M2 terminology does not reflect sequential and pleiotropic characteristics of infiltrating macrophages [49]. Ly6C^+^ or MHC^−^ macrophages that first arrive at the injury site and the Ly6C^−^ or MHC^+^ macrophages present in the later phases of skeletal muscle regeneration may represent M1-­biased and M2-­biased populations, respectively [50, 51]. M1 macrophages contribute to a spectrum of pro-inflammatory cytokines, including, but not limited to, IFN-γ, TNF-α, IL-1β and IL-6, conditioning the immune microenvironment to promote the rapid proliferation of MuSCs and simultaneously restrain the early differentiation steps of MuSCs [48, 52–56]. Moreover, these pro-inflammatory macrophages, through their expression of TNF-α, directly induce apoptosis of FAPs, thus regulating their number and ultimately preventing pathological muscle fibrosis [57]. Macrophage-derived niche signals are vital for MuSC proliferation. The ‘dwelling’ macrophages, a specific subset of macrophages within the injury area, secrete nicotinamide phosphoribosyltransferase (NAMPT), which, as mitogenic stimuli, activates MuSCs through CCR5 [58]. However, the variety and concentration of inflammatory factors produced by myeloid cells cannot meet the needs of extensive expansion of MuSCs. Hence, T cells play an important role in conditioning the endogenous inflammatory microenvironment and maintaining exponential growth of MuSCs. Specifically, the cocktail of four cytokines (IL-1α, IL-13, IFN-γ and TNF-α) secreted from activated T cells assures MuSC expansion in *Rag1*^–/–^ mice that lack T cells but have intact macrophages, and promoted the proliferation of undifferentiated MuSCs through 20 passages in vitro [59]*.* The addition of these four cytokines in culture medium, mimicking the endogenous inflammatory microenvironment, presents a promising method to efficiently expand MuSCs without loss of self-renewing potency, and breaks the bottleneck that had limited long-term serial expansion of functional MuSCs to meet the demands for clinical therapeutic applications [60].

## Regulation of myogenesis by anti-inflammatory responses

The resolution of inflammation, associated with the transition from the pro-inflammatory to the anti-inflammatory phase, is functionally coupled to the transition of regeneration states from the proliferative stage to the differentiation and growth stage of myogenesis and is required for tissue recovery [28]. M2 macrophages are crucial in avoiding detrimental inflammation and returning to tissue homeostasis. They contribute to myogenesis, angiogenesis, and ECM remodeling through reciprocal interactions with surrounding cells, including MuSCs, endothelial cells and FAPs [48, 50, 57, 61, 62]. Advances have recently been made in the understanding of the mechanisms governing the spatiotemporal phenotype shift of macrophages. Various metabolic programs can control macrophage inflammatory status [63, 64]. Mass spectrometry-based lipidomics coupled with transcriptomics identified a temporal shift of lipid metabolism, from pro-inflammatory lipid mediators (e.g., leukotrienes, prostaglandins) to specialized pro-resolving lipid mediators (e.g., resolvins and lipoxins), in the transition from Ly6C^+^ macrophages to Ly6C^−^ macrophages [65]. In particular, activation of the metabolic regulator AMP-activated protein kinase α1 (AMPKα1) is required for the shift of macrophage polarization. Myeloid-specific deletion of AMPKα1 using lysozyme M-Cre (LysM-Cre) mouse strain resulted in exacerbated muscle damage and delayed repair/regeneration while infiltrating macrophages during the first phase failed to skew from a pro-inflammatory to an anti-inflammatory phenotype at the time of resolution of inflammation [66]. Insulin growth factor 1 (IGF1) possesses pleiotropic biological functions in M1 macrophages. On the one hand, it exerts paracrine action on activated MuSCs to facilitate their proliferation. On the other hand, myeloid cell-­derived IGF1 also influences the outcomes of muscle regeneration through an autocrine effect, which regulates the transition of infiltrating macrophages from a M1 to a M2 phenotype [67, 68]. Prostaglandin E2 (PGE2), another important immunosuppressive factor, can inhibit the proliferation of T cells, reduce the toxic effects of NK cells, and shape the functions and phenotypes of macrophages [69–71]. In addition, PGE2 also acts as a strong mitogen for MuSCs, through binding to the EP4 receptor on their own surface, triggering the intracellular cAMP/phosphor-CREB pathway and activating the proliferation-inducing transcription factor *Nurr1*, ultimately robustly augmenting MuSC expansion [72]. Regulatory T (Treg) cells are also crucial for returning to the homeostasis of injury muscles [73, 74]. Depletion of Treg cells slowed regeneration, prolonged inflammation and perturbed the expression of myogenic transcription factors while the accrual and the polarization towards the M2 phenotype of muscle macrophages in vivo could not be completed [51, 75, 76].

## Mechanisms of immunomodulation by MuSCs

Muscle regeneration involves highly coordinated cellular responses between different varieties of immune cells and MuSCs. Disturbance of the coordination between the different types of cells will lead to delay or even failure of muscle repair. The synchronized functional changes of trafficking macrophages during skeletal muscle regeneration directly determine MuSC fate and myogenesis progress. Inflammatory factors released from activated T cells in high concentrations promote MuSCs to enter the cell cycle and rapidly proliferate, thus ensuring plenty of stem cell reserves for muscle regeneration. Mounting evidence has confirmed the critical roles of immune cells in directing the fate of MuSCs. However, it is unclear whether there is a bidirectional interaction between immune cells and MuSCs so that MuSCs can in turn regulate the functions of immune cells.

Skeletal muscle is not only an important endocrine tissue, but also one of the main immunomodulatory organs [27]. Myocytes, non-professional antigen-presenting cells, actively participate in the functional regulation of immune cells under physiological and pathological conditions. Myocytes participate in the innate immune response and affect adaptive immunity through secreting myokines [77]. Muscle trauma is accompanied by prominent myocyte necroptosis, in which tenascin-C released by necroptotic myofibers promotes MuSC proliferation to facilitate muscle regeneration [78]. Beside myocytes, do MuSCs, the main myogenic cells in the damaged tissue microenvironment endowed mitogenic functions of necroptosis and pro-inflammatory cytokines, also possess immunomodulatory properties?

**Fig. 2:**
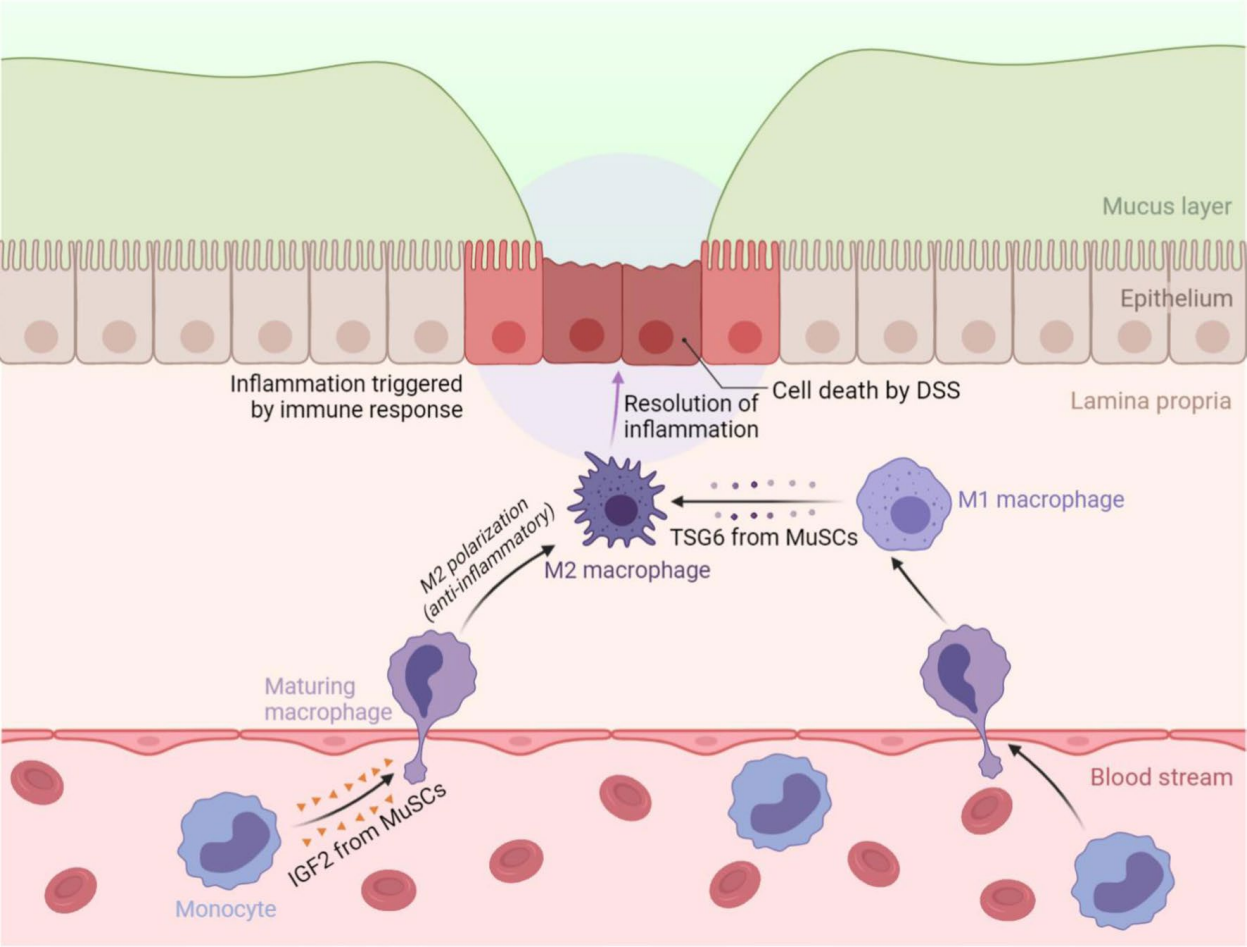
The immunomodulatory properties of MuSCs during colon inflammation. The resolution of inflammation allows tissue to return to homeostasis. The immunomodulatory properties of MuSCs are required from this process. On the one hand, MuSCs can act on maturing macrophages and confer them with anti-inflammatory properties via IGF2 secretion, thus ameliorating DSS-induced colitis and facilitating tissue repair. On the other hand, inflammatory cytokines-licensed MuSCs, particularly with IFN-γ and TNF-α, can also alleviate DSS-induced colitis through IDO metabolites-mediated TSG6 production, which mediates the switch of macrophages from a M1 to a M2 phenotype

We have recently addressed this question experimentally using an inflammatory bowel disease (IBD) model in vivo (Fig. 2). We identified a novel anti-inflammatory function of MuSCs that is based on their production of IGF2. By endowing maturing macrophages an oxidative phosphorylation (OXPHOS)-dependent anti-inflammatory property, the IGF2-producing MuSCs can thus orchestrate an anti-inflammatory microenvironment that favors tissue repair and regeneration in colon tissues [79]. TNF-stimulated gene 6 (TSG6), an important immunoregulatory factor, can suppress inflammation by inducing pro-inflammatory macrophages to adopt an anti-inflammatory phenotype in LPS-induced acute lung injury and dextran sulfate sodium (DSS)-induced colitis model [80, 81]. MuSCs stimulated by inflammatory factors IFN-γ and TNF-α exhibit an anti-inflammatory function that depends on the indoleamine 2,3-dioxygenase (IDO)-TSG6 axis in IBD mice. Mechanistically, the IDO metabolites kynurenine (KYN) or kynurenic acid (KYNA) promote TSG6 expression through activating aryl hydrocarbon receptor (AHR) signaling [82]. Thus, MuSCs do not only respond to inflammatory cues in damage microenvironment but also act on immune cells to shape immune microenvironment to facilitate tissue repair. The new discovery of MuSCs-based immunomodulatory ability expands the potential applications of stem cell therapies in pathological conditions beyond muscle-related diseases.

## New modes of MuSC-based therapy

There has been a long-standing debate about stem cell-based regeneration and repair mechanism, is it based on cell replacement or cell empowerment. Indeed, only few transplanted stem cells are capable of differentiating into daughter cells at the damaged site or even persisting for a sufficiently long period in vivo [83]. In addition, the results from animal experiments of xenotransplantation and preclinical studies of allogeneic stem cell transplantation have clearly demonstrated therapeutic potentials of stem cell ‘decellularization’, thus strongly supporting cell empowerment as the paradigm of stem cell therapy. Stem cells ‘licensed’ by inflammatory factors can dictate the phenotypes and functional metamorphoses of immune cells by producing immunoregulatory factors, and guide the outcomes of inflammation in various diseases. Mesenchymal stem cells (MSCs) can unleash powerful therapeutic effects under different kinds of inflammatory conditions via cell empowerment [84, 85]. In our study, we found that the MuSC secretome possessed a therapeutic efficacy equivalent to that of MSCs in IBD mice, thus indicating that MuSCs can promote tissue repair indirectly through cell empowerment [79]. It was also demonstrated that MuSCs’ exosomes could prevent excessive ECM deposition and facilitate muscle regeneration via regulating collagen expression in fibrogenic cells [86]. Further validation of cell empowerment or promotion of tissue homeostasis by stem cells in physiological and pathological conditions will advance our understanding of MuSC biology and expand the application scope of MuSCs.

IGF2, a strong mitogen for MuSCs [87], is a vital immunomodulatory mediator that confers maturing macrophages anti-inflammatory properties [79, 88, 89]. The actions of IGF2 are coupled temporally and spatially to the myogenesis program between MuSC proliferation and immunomodulation. During the early stage of muscle regeneration, MuSCs produce IGF2 to promote self-proliferation and expansion. However, along with the mounting number of MuSC populations, the local concentration of IGF2 in tissue microenvironments becomes sufficiently high to determine the inflammatory phenotypes of infiltrating immune cells. In this scenario, the tissue microenvironment completes the transition from a pro-inflammatory to an anti-inflammatory phase with the assistance of MuSCs. The resolution of inflammation is required for tissue recovery, in which MuSCs stop proliferation and enter the differentiation process. Therefore, in the early stage of muscle regeneration, IGF2 acts as a ‘throttle’ of MuSC proliferation response, while in the late stage of muscle regeneration, abundant IGF2 functions as the ‘brake’ of MuSC proliferation response through inducing resolution of inflammation (Fig. 1). The distinct actions of IGF2 in different phases ensure that those controllable MuSC populations can be fully applied to the subsequent muscle differentiation process, avoiding excessive accumulation of activated MuSCs in local tissue, and improving the application of stem cell during tissue regeneration.

The immunomodulatory ability of MuSCs represents a new mechanism of action by stem cells in the treatment of muscle-related diseases. Immunological and inflammatory processes downstream of dystrophin deficiency contribute to muscle pathology in duchenne muscular dystrophy (DMD). Modulating the inflammatory response and inducing immunological tolerance to de novo dystrophin expression are critical to the success of dystrophin-replacement therapies [90]. The transplantation of MuSCs from healthy donors to DMD patients is based on the assumption that nascent muscle will be formed from the transplanted stem cells. Our findings suggest that MuSCs may also function in taming inflammation in damaged muscle. MuSC-mediated immunomodulation could be of continued importance in completing normal differentiation program and achieving successful dystrophin-replacement therapy. In addition, the immunomodulatory ability of MuSCs provides new potential venues in the treatment of inflammatory diseases by MuSCs. MuSC secretome as a new therapeutic agent can greatly reduce allograft rejection reaction of treated patients, while reducing safety and ethical risks, and facilitate therapeutic effects of stem cells in various inflammatory diseases.

## A novel therapeutic use in the COVID-19 pandemic

The pandemic of coronavirus disease 2019 (COVID-19), caused by severe acute respiratory syndrome coronavirus 2 (SARS-CoV-2), has caused over 4 million deaths up to the present. SARS-CoV-2 infection results in a broad spectrum of clinical manifestations, including both reproductive and neurological implications [91–94]. Indeed, COVID-19 is characterized by excessive production of pro-inflammatory cytokines and acute lung damage associated with patient mortality [95–100]. Various clinical trials based on the potent immunoregulatory ability of MSCs were launched in different countries in various cases with COVID-19 infection. MSC administration appeared to be efficacious in ameliorating over-activated inflammation, contributing to recovery from lung damage, preventing long-term pulmonary disability, and reducing mortality. In addition, MSC infusion in COVID-19 patients had excellent safety [101–108] (Table 1). In this scenario, the therapeutic efficacy of MuSCs equivalent to that of MSCs can be applied in the suppression of hyperactive immune response and promotion of tissue repair, as in DSS-induced IBD.

**Table 1:** Clinical studies of MSC treatment for COVID-19 patients

Trial ID	Trial design	Indications	MSC source	Dose and routes of MSC administration	Number of patients	Clinical outcomes	Refs
ChiCTR2000029990	Phase 1, open-label, single-center, case–control	Moderate/Severe/Critical	Clinical grade MSCs	1 × 10^6^ cells/kg, i.v	10	The pulmonary functions and symptoms were significantly improved after MSC infusion.	101
ChiCTR2000031494	Phase 1, open-label, randomized, standard treatment–control	Severe/Critical	Umbilical cord	2 × 10^6^ cells/kg, i.v	41	MSC infusion showed improved clinical manifestations.	102
NCT04252118	Phase 1, open-label, single-center, case–control	Moderate/Severe	Umbilical cord	3 × 10^7^ cells/dose, i.v	18	Intravenous MSC infusion in moderate and severe COVID-19 patients was safe and well-tolerated.	103
NCT04269525	Phase 2, case–control	Severe/Critical	Umbilical cord	1 × 10^8^ cells/dose, i.v	16	MSC infusion showed improvement in ventilatory, radiological and biological parameters. No MSC infusion related adverse or allergic reactions and no delayed hypersensitivity or secondary infections were reported.	104
NCT04288102	Phase 2, randomized, double-blind, placebo-control	Severe	Umbilical cord	4 × 10^7^ cells/dose, i.v	100	MSC infusion was safe and exerted improvement in total lung lesion proportion and solid component lesion. There was an increased 6-min walking distance in MSC group.	105
NCT04348461	Phase 1, prospective nonrandomized open-label cohort	Severe/Critical	Adipose tissue	1 × 10^6^ cells/kg, i.v	13	No MSC infusion-associated adverse events were reported. Improvement in ventilatory, radiological and biological parameters was associated with clinical status.	106
NCT04355728	Phase 1/2a, randomized, double-blind	ARDS	Umbilical cord	10 ± 2 × 10^7^ cells/dose, i.v	24	MSC infusion was safe. The levels of pro-inflammatory cytokines were significantly decreased, and patient survival and recovery time were improved.	107
IRCT20200217046526N2	Phase 1	ARDS	Umbilical cord and placental	2 × 10^8^ cells/dose, i.v	11	MSC infusion in critical illness COVID-19 patients was safe and well-tolerated. MSC group had improved respiratory distress and reduced inflammatory signatures.	108

*MSC,* mesenchymal stem cell; *i.v.,* intravenous injection; *ARDS,* acute respiratory distress syndrome

## Perspective

As mentioned above, MuSCs do not only have well-known muscle differentiation functions, but also possess important immunomodulatory properties. The relationship between MuSCs and immune cells is bidirectional. In addition to passively receiving inflammatory cues from immune cells for proliferation and differentiation, MuSCs can also actively secrete immunomodulatory factors to induce the resolution of inflammation. Based on the immunoregulatory effect of stem cells, more than 1000 clinical disease treatment studies of MSCs have been carried out around the world. However, there are only dozens of cases of clinical research involving MuSCs, and they are fully focused on the treatment of skeletal muscle diseases based on the muscle differentiation function of MuSCs. The research on the immunoregulatory function of MuSCs will further broaden the application fields of MuSCs. MuSCs are expected to become one of the important stem cell types for the treatment of inflammatory diseases in the foreseeable future.

## Data Availability

Not applicable.
